# Sensory Perception of an Oral Rehydration Solution during Exercise in the Heat

**DOI:** 10.3390/nu13103313

**Published:** 2021-09-23

**Authors:** Olivia Kitson, Kay Rutherfurd-Markwick, Andrew Foskett, Jason Kai Wei Lee, Charles Diako, Marie Wong, Ajmol Ali

**Affiliations:** 1 School of Sport, Exercise and Nutrition, Massey University, Auckland 0745, New Zealand; livvykitson@gmail.com (O.K.); a.foskett@massey.ac.nz (A.F.); 2 School of Health Sciences, Massey University, Auckland 0745, New Zealand; K.J.Rutherfurd@massey.ac.nz; 3 Centre for Metabolic Health Research, Massey University, Auckland 0745, New Zealand; 4 Human Potential Translational Research Programme, Yong Loo Lin School of Medicine, National University of Singapore, Singapore S119228, Singapore; phsjlkw@nus.edu.sg; 5 Department of Physiology, Yong Loo Lin School of Medicine, National University of Singapore, S117593, Singapore; 6 Global Asia Institute, National University of Singapore, S119076, Singapore; 7 N.1 Institute for Health, National University of Singapore, S117456, Singapore; 8 Institute for Digital Medicine, National University of Singapore, S117456, Singapore; 9 Singapore Institute for Clinical Sciences, Agency for Science, Technology and Research (A*STAR), S117609, Singapore; 10 School of Food and Advanced Technology, Massey University, Auckland 0745, New Zealand; c.diako@massey.ac.nz (C.D.); M.Wong@massey.ac.nz (M.W.)

**Keywords:** dehydration, electrolytes, palatability, saltiness, sports drinks, thirst

## Abstract

Prolonged exercise in the heat elicits a number of physiological changes as glycogen stores are low and water and electrolytes are lost through sweat. However, it is unclear whether these changes provoke an increase in liking of saltiness and, therefore, palatability of an oral rehydration solution (ORS). Twenty-seven recreationally active participants (*n* = 13 males; *n* = 14 females) completed sensory analysis of an ORS, a traditional sports drink (TS), and a flavored water placebo (PL) at rest and during 60 min (3 × 20-min bouts) of cycling exercise at 70% age-predicted maximum heart rate (HR_max_) at 35.3 ± 1.4 °C and 41 ± 6% relative humidity. Before and after every 20 min of exercise, drinks were rated (using 20-mL beverage samples) based on liking of sweetness, liking of saltiness, thirst-quenching ability, and overall liking on a nine-point hedonic scale. Hydration status was assessed by changes in semi-nude body mass, saliva osmolality (S_Osm_), and saliva total protein concentration (S_PC_). After 60 min of exercise, participants lost 1.36 ± 0.39% (mean ± SD) of body mass and there were increases in S_Osm_ and S_PC._ At all time points, liking of sweetness, saltiness, thirst-quenching ability, and overall liking was higher for the TS and PL compared to the ORS (*p* < 0.05). However, the saltiness liking and thirst-quenching ability of the ORS increased after 60 min of exercise compared to before exercise (*p* < 0.05). There was also a change in predictors of overall liking with pre-exercise ratings mostly determined by liking of sweetness, saltiness, and thirst-quenching ability (*p* < 0.001), whereas only liking of saltiness predicted overall liking post-exercise (R^2^ = 0.751; *p* < 0.001). There appears to be a hedonic shift during exercise in which the perception of saltiness becomes the most important predictor of overall liking. This finding supports the potential use of an ORS as a valuable means of hydration during the latter stages of prolonged and/or intense exercise in the heat.

## 1. Introduction 

Voluntary dehydration occurs when ad libitum fluid intake is insufficient to match fluid losses, leading to a cumulative loss of body water [1]. Threshold decreases in blood volume (hypovolemia) and increases in plasma osmolality (hyperosmolality), usually stimulating thirst and subsequent fluid intake [2,3]. Hypohydration with a body mass loss of ≥2% is often associated with severe impairments in thermoregulatory, metabolic, and cardiovascular functions, often leading to adverse effects on performance and health [4,5,6]. During exercise, however, thirst becomes an unreliable stimulus to drinking and is often alleviated before complete rehydration is achieved [7,8]. Indeed, athletes typically lose 1–2% of their body mass during exercise despite fluids being freely available [1,9]. 

Sports drinks are designed to promote hydration before, during and after exercise; they exist in a variety of formulations with water, carbohydrate (CHO; typically 6–9% but with some containing lower < 6%), and electrolytes, with sodium (Na^+^) being the main ingredient in particular [10]. Carbohydrate is added to provide sweetness and energy; however, the greater the concentration of CHO, the slower the rate of gastric emptying and higher the risk of gastrointestinal distress [11]. In addition, the electrolyte content of traditional sports drinks (22–25 mmol/L Na) is insufficient to match Na losses through sweat for many endurance athletes [1]. Sodium is an essential electrolyte which functions to maintain plasma osmolality and stimulate thirst [12]. For these reasons, oral rehydration solutions (ORSs) with relatively higher Na (30–90 mmol/L) and lower CHO concentrations (2–3% CHO) may be more effective for promoting hydration, particularly when sweat losses are large [13]. Due to poor palatability ratings, however, ORSs are generally not used in an exercise context [12]. Nevertheless, Fan et al. [14] recently showed that an ORS was more effective at restoring fluid deficit during recovery from exercise than sports drinks or water without compromising the drink’s palatability with increased sodium concentration.

Beverage palatability has been identified as a key determinant of voluntary fluid intake with food and fluid items being much more likely to be consumed if they are perceived as being pleasant [15,16]. Beverage temperature, carbonation level, and flavor all contribute to the overall hedonic experience, with cool (<15 °C) [17], flavored [18], and non-carbonated [19] beverages being the most palatable during exercise and thus, consumed in greater quantities [15]. However, there appears to be a hedonic shift during exercise in which food and fluid items which are least liked at rest can progressively increase in perceived pleasantness [15]. 

Prolonged exercise elicits an increase in requirements for water, CHO, and electrolytes, thus an increase in pleasantness of various food and fluid items has been attributed to their physiological usefulness perceived by the consumer [15,20]. An increase in liking of low osmolality fluids immediately after exercise has been attributed to the importance of maintaining body fluid balance [21]. Alternatively, increases in perception and liking of sweetness have been attributed to the role of CHO in energy provision during exercise [22,23]. Most importantly, the physiological usefulness theory has been proposed to explain an increase in salt preference or ‘Na^+^ appetite’ with exercise, especially in those exhibiting large sweat losses [24,25,26]. Given that sweat and salt losses are exacerbated when exercise is performed in the heat, such environments may favor ‘Na appetite’.

Compared to traditional sports drinks, ORSs have a relatively high intensity of saltiness contributing to poor palatability ratings at rest. Only one study has tested the palatability of an ORS throughout exercise in the heat [14], but palatability was assessed across trials with different solutions and using only a small sample size (*n* = 12). Therefore, the aim of this study was to test the palatability of an ORS at various stages of exercise in the heat in recreational exercisers. 

## 2. Materials and Methods

### 2.1. Participants

Twenty-seven recreationally active participants (*n* = 13 males; *n* = 14 females; mean ± SD; age 25 ± 8.0 years, height 172.2 ± 8.5 cm, body mass 69.8 ± 8.9 kg) volunteered to take part in this study. All participants were recruited on the basis that they were capable of performing 60 min of cycling exercise (3 × 20-min bouts) in a warm room (35.3 ± 1.4 °C, RH 41 ± 6%) at a moderate intensity (70–80% HR_max_) whilst wearing a sweat suit to promote fluid loss. Participants were provided with an information sheet, and following completion of a health screening questionnaire, written informed consent was obtained. Participants were excluded if they had a heart condition, took prescription drugs, experienced chest pain while exercising, or had any other issues that may have prevented them from completing 60 min of moderate intensity exercise in the heat. All protocols and procedures had prior approval by the local human ethics committee. 

### 2.2. Familiarisation Procedures 

At least 24 h prior to the main trial, participants were required to attend the laboratory for a familiarization session. Following height (stadiometer; Surgical and Medical Products, Auckland, NZ, USA) and body mass (using digital scales; A&D Weighing Hv 200-KGL, Adelaide, Australia) measurements, participants were familiarized with the sensory protocol and methods for collecting urine and saliva samples. Participants tried on the sweat suit and then performed a short (5 min) incremental cycle to determine the appropriate load equivalent to 70–80% age-predicted HR_max_.

### 2.3. Study Design and Procedures

**Figure 1:**
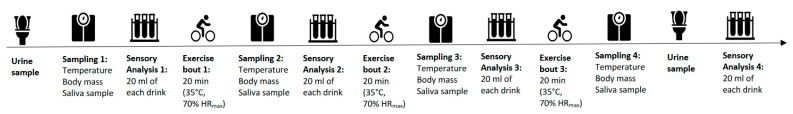
Overview of the exercise and sensory protocol comprised 3 × 20-min bouts of cycling exercise in a warm room (30–35 °C) at a moderate intensity (70–80% HR_max_). Three drinks: an oral rehydration solution (ORS), a beverage formulation based on a traditional sports drink (TS), and a flavored water placebo (PL) were evaluated for liking of saltiness, sweetness, thirst-quenching ability, and overall liking on 9-point scales before and after each 20-min exercise bout. Urine samples were collected before the 60 min of exercise; and saliva samples, aural temperature, and semi-nude body mass were assessed before and after each 20-min exercise bout. Each sensory analysis and sampling was completed within 10 min prior to the subsequent exercise bout.

Participants came into the laboratory for a single main trial after refraining from caffeine, alcohol and physical activity for at least 24 h prior. Figure 1 shows a schematic representation of the overall study design. Participants wore a sweat suit during exercise and (in a private room) removed the suit, towel dried themselves, before undertaking body mass, saliva, and sensory analyses. Sensory evaluation was conducted in a single trial, of an ORS, a traditional sports drink, and a flavored water placebo at rest and after each of 3 × 20-min bouts of exercise in a randomized, double-blind design. Urine samples were collected before and after the 60 min of exercise; and saliva samples, aural temperature, and semi-nude body mass were assessed before and after each 20-min exercise bout. After the final exercise bout, a urine sample was collected. Participants were then able to take a shower and rehydrate with refreshments provided.

### 2.4. Exercise Measures

Participants completed 3 × 20-min cycling bouts at the predetermined load and speed (70–80% of age-predicted HRmax) in a warm room (temperature 35.3 ± 1.4 °C, humidity 41 ± 6%) while wearing a sweat suit to promote fluid loss. During exercise, heart rate was recorded every 5 min via short-range telemetry (Polar Electro S610i, Polar, Kempele, Finland). After each 20-min exercise bout, aural temperature and perceptual scales (ratings of perceived exertion (RPE) [27], a felt arousal scale (FAS) [28], and a feeling scale (FS) [29], thermal comfort) were assessed. 

### 2.5. Drinks for Sensory Analysis 

Three drinks of varying osmolarity, electrolyte content, and energy content per liter (Table 1) were used in this study: an oral rehydration solution (ORS; composition based on WHO recommendations of osmolarity between 200–310 mmol/L and other requirements such as electrolyte content), a beverage formulation based on a traditional sports drink (TS), and a flavored water placebo (PL). Drinks were prepared in a food technology laboratory on campus, pasteurized, hot filled, and then stored in a chiller at 4 °C. All drinks were colorless and non-carbonated, with the same concentration of mixed berry flavoring (0.8 g/L; Symrise Asia Pacific Pte, Singapore). 

### 2.6. Urine and Saliva Sampling 

Urine samples were used to determine osmolality using an osmometer (Astori Technica- Osmotouch 1 osmometer, Poncarale, Italy), urine specific gravity using a refractometer, and color using an 8-point color scale [30]. Saliva samples, collected using the bud method [31], were used to determine osmolality using an osmometer and protein content using the Bradford method [32]. 

### 2.7. Sensory Analysis 

Liking of sweetness, saltiness, thirst-quenching ability as well as overall liking for each of the three drinks were evaluated before and after each 20-min exercise bout on a 9-point hedonic scale with 1 = dislike extremely and 9 = like extremely. In a private room, participants were presented with 20 mL of each fluid in clear and 25 mL of sample cups labelled with a 3-digit random code. The samples were evaluated in a randomized order. Data were captured using Compusense^®^ Cloud (Guelph, ON, Canada) on iPads (Apple Inc., Cupertino, CA, USA). Participants were required to consume the entire sample and cleanse their palate with filtered water between samples. The palate cleansing water was expectorated into a cuspidor. A 30-s break was allowed between sample tasting for palate cleansing. These were weighed afterwards to ensure participants did not consume the filtered water. 

### 2.8. Statistical Analysis 

Statistical analysis was conducted using IBM SPSS Statistics (Version 24.0. Armonk, NY, USA: IBM Corp). Sensory data were analyzed using a two-way analysis of variance (ANOVA) with repeated measures to compare each treatment (ORS, PL, TS) across each time point (0, 20, 40, 60 min of exercise). Mauchly’s test of sphericity was used to determine whether the assumption of sphericity was being violated by the data. Where this did occur, the Huynh–Feldt correction was applied. Physiological responses were analyzed using a one-way ANOVA for parametric data and Wilcoxon signed rank-tests were performed for non-parametric data. Where a main effect or interaction effect was observed, a pairwise post-hoc analysis using the Holm–Bonferroni adjustment was performed to determine specific differences. Levene’s test was used to test the assumption for homogeneity of variance. Relationships between variables were tested using the Pearson’s correlation coefficient for parametric data and Spearman’s correlation for non-parametric data and interpreted as “weak, r = 0.1”, “moderate, r = 0.3”, and “strong, r = 0.7” [33]. Multiple linear regression analyses were conducted to determine the key predictors of overall liking. Cohen’s d was calculated to measure the magnitude of the findings and interpreted using Cohen’s [34] guidelines for effect sizes as “small, d = 0.2”, “medium, d = 0.5”, and “large, d = 0.8”. Parametric data are presented as mean ± standard deviation (SD), log-transformed data as geometric mean (95% confidence intervals; CI), and non-parametric data as median (25, 75 percentiles). The significance level was *p* < 0.05 for all tests. 

## 3. Results 

### 3.1. Physiological and Perceptual Responses to Exercise 

Physiological responses throughout stages of exercise are reported in Table 2. After 60 min of exercise, participants achieved a mean (±SD) body mass loss (BML) of 0.94 ± 0.30 kg (1.36 ± 0.39%). From 0 min to 60 min of exercise, there was an increase in mean (95% CI) saliva osmolality (S_Osm_) from 85 (78, 92 mOsmol/kg to 113 (102, 124) mOsmol/kg (*p* < 0.001) and saliva total protein concentration (S_PC_) from 1.27 (1.06, 1.49) mg/mL to 3.17 (2.51, 3.96) mg/mL (*p* < 0.001). Heart rate (*p* < 0.001), aural temperature (*p* = 0.008), ratings of perceived exertion (*p* < 0.001), feeling scale (*p* < 0.001), and thermal comfort (*p* < 0.001) all increased throughout the 60 min of exercise. Percent BML showed a moderate positive correlation with S_PC_ (r = 0.353, *p* = 0.001) and S_Osm_ (r = 0.375, *p* = 0.001). Percent BML showed a moderate negative correlation with ratings of saltiness liking (r = −0.316, *p* = 0.004), thirst-quenching ability (r = −0.323, *p* = 0.003), and overall liking of fluids (r = −0.234, *p* = 0.036), but no relationship with sweetness liking (r = −0.183, *p* = 0.102). 

### 3.2. Sensory Analyses

#### 3.2.1. Liking of Saltiness

**Figure 2:**
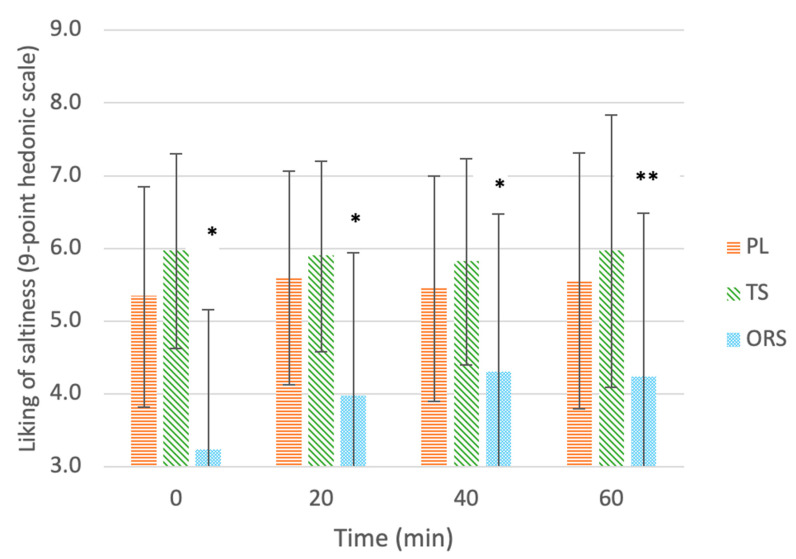
Liking of saltiness of drinks: ORS—oral rehydration solution, PL—placebo, TS—traditional sports drink, on a 9-point hedonic scale throughout 60 min of exercise. Values are mean ± SD. * Significant effect of the treatment vs. the placebo (*p* < 0.05). ** Significant delta change in ratings compared to 0 min before exercise (*p* < 0.05).

Figure 2 shows the mean (± SD) ratings of saltiness liking for each drink after each exercise time. At all time points, liking of saltiness differed between drinks (main effect of treatment, *p* < 0.001) and was higher for PL (5.48 ± 1.58, *p* = 0.012, d = 0.84) and TS (5.91 ± 1.49, *p* < 0.001 d = 1.09) than ORS (3.93 ± 2.09), while there were no differences between PL and TS (*p* < 0.05, d = 0.28). There was no main effect of exercise time (*p* = 0.109) and no interaction between drink type and exercise time (*p* = 0.199). Saltiness ratings differed between drinks throughout exercise (χ^2^(2) = 7.043, *p* = 0.030), specifically median (25, 75 percentiles) percent change in saltiness ratings for the ORS, PL, and TS were 20% (0, 100%), 0% (−16.7, 20%), and 0% (−22.2, 20%), respectively. There was an increase in liking of saltiness for ORS vs. PL (Z = −2.341, *p* = 0.019) and for ORS vs. TS (Z = −2.386, *p* = 0.017) but there was no difference between PL and TS (Z = −0.382, *p* = 0.702). 

#### 3.2.2. Liking of Sweetness 

**Figure 3:**
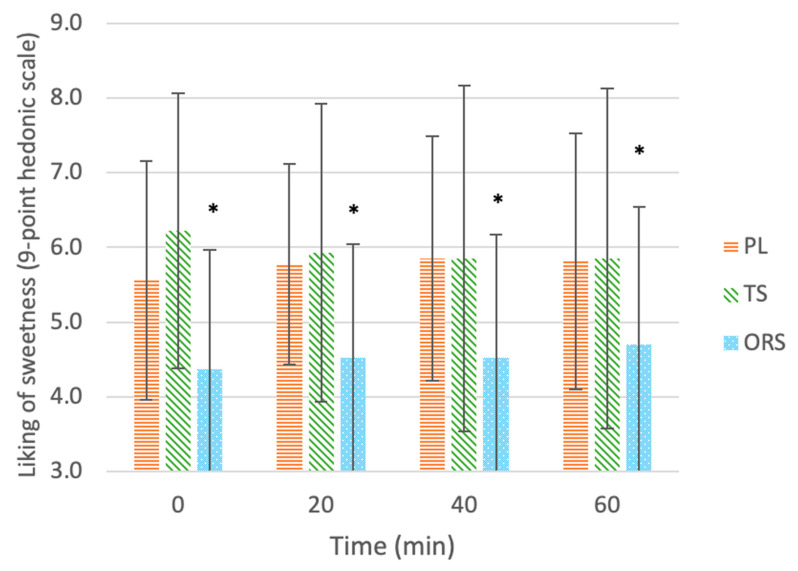
Liking of sweetness of drinks: ORS—oral rehydration solution, PL—placebo, TS—traditional sports drink, on a 9-point hedonic scale throughout 60 min of exercise. Values are mean ± SD. * Significant effect of the treatment vs. the placebo (*p* < 0.05).

Figure 3 shows the mean (± SD) ratings of sweetness liking for each drink after each exercise time. At all time points, liking of sweetness differed between drinks (main effect of treatment, *p* = 0.003) and was higher for PL (5.75 ± 1.57, *p* = 0.09, d = 0.76) and TS (5.96 ± 2.11, *p* = 0.015, *d* = 0.76) than ORS (4.53 ± 1.65), while there were no differences between PL and TS (*p* < 0.05, *d* = 0.11). There was no main effect of exercise time (*p* = 0.985) and no interaction between drink type and exercise time (*p* = 0.732). There was no difference in percent change in sweetness ratings between drinks throughout exercise (χ^2^(2) = 3.293, *p* = 0.193); median (25, 75 percentiles) perceived sweetness for the ORS, PL, and TS were 0% (−20, 25%), 0% (0, 33.3%), and 0% (−16.7, 28.6%), respectively.

#### 3.2.3. Thirst-Quenching Ability 

**Figure 4:**
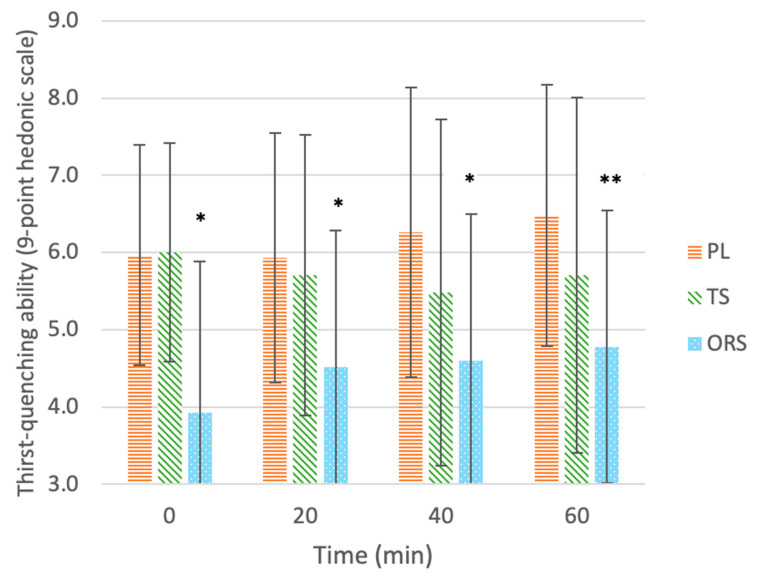
Liking of thirst-quenching ability of drinks: ORS—oral rehydration solution, PL—placebo, TS—traditional sports drink, on a 9-point hedonic scale throughout 60 min of exercise. Values are mean ± SD. * Significant effect of the treatment vs. the placebo (*p* < 0.05). ** Significant delta change in ratings compared to 0 min before exercise (*p* < 0.05).

Figure 4 shows the mean (± SD) ratings of thirst-quenching ability for each drink after each exercise time. At all time points, thirst-quenching ability differed between drinks (main effect of treatment, *p* = 0.001); ratings of thirst-quenching ability were higher for PL (6.2 ± 1.7, *p* = 0.002, d = 0.98) and TS (5.7 ± 1.9, *p* = 0.025, d = 0.67) than ORS (4.4 ± 1.9), while there were no differences between PL and TS (*p* > 0.05, d = 0.24). There was no main effect of exercise time (*p* = 0.336) and no interaction between drink type and exercise time (*p* = 0.151). There was a significant difference in percent change in thirst-quenching ability ratings between drinks throughout exercise (χ^2^(2) = 7.327, *p* = 0.026); the median (25, 75 percentiles) percent change in thirst-quenching ability ratings for the ORS, PL and TS were 50% (0, 66.7%), 0% (−14.3, 28.6%), and 0% (−25, 14.3%), respectively. There was an increase in percent thirst-quenching ability for ORS vs. PL (Z = −2.046, *p* = 0.041) and for ORS vs. TS (Z = −2.681, *p* = 0.007). There was also a trend for a difference between PL and TS (Z = −1.925, *p* = 0.054). 

#### 3.2.4. Overall Liking 

**Figure 5:**
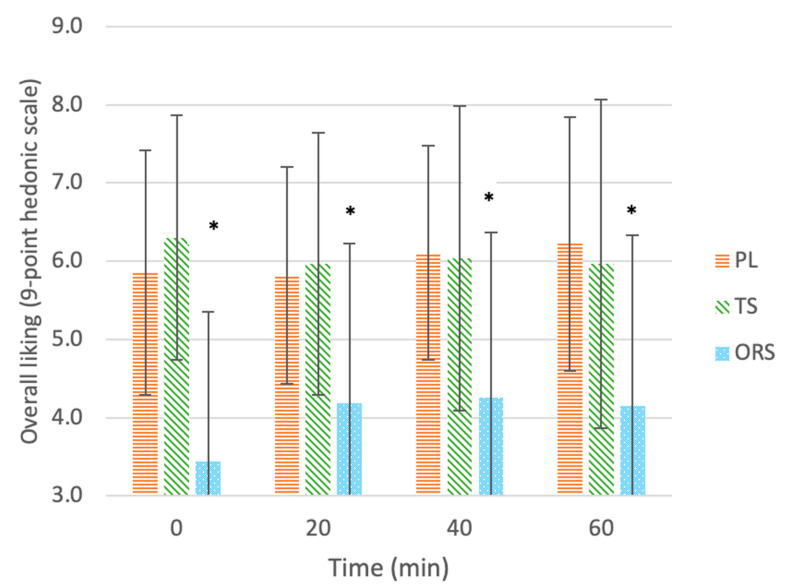
Overall liking of drinks: ORS—oral rehydration solution, PL—placebo, TS—traditional sports drink, on a 9-point hedonic scale throughout 60 min of exercise. Values are mean ± SD. * Significant effect of the treatment vs. the placebo (*p* < 0.05).

Figure 5 shows the mean (± SD) ratings of overall liking for each drink after each exercise time. At all time points, ratings of overall liking differed between drinks (main effect of treatment, *p* < 0.001); overall liking was higher for PL (6.0 ± 1.5, *p* = 0.001, d = 1.1) and TS (6.1 ± 1.8, *p* < 0.001, d = 1.06) than ORS (4.0 ± 2.1), while there were no differences between PL and TS (*p* > 0.05, d = 0.04). There was no main effect of exercise time (*p* = 0.353) and no interaction between drink type and exercise time (*p* = 0.251). There was no difference in percent change in overall liking ratings between drinks throughout exercise (χ^2^(2) = 4.989, *p* = 0.083). The median (25, 75 percentiles) percent change in ratings of overall liking for the ORS, PL and TS were 0% (0, 100%), 0% (−12.5, 40%), and 0% (−20, 0%), respectively. 

#### 3.2.5. Predictors of Overall Liking 

Liking of sweetness, saltiness, and thirst-quenching ability were identified as the main predictors of overall liking for all drinks pre-exercise (R^2^ = 0.850; *p* < 0.001). However, only liking of saltiness was identified as a predictor of overall liking post-exercise (R^2^ = 0.751; *p* < 0.001). For the ORS, overall liking was mainly determined by liking of sweetness and saltiness pre-exercise (R^2^ = 0.837; *p* < 0.001), whereas liking of saltiness and thirst-quenching ability were the most important predictors post-exercise (R^2^ = 0.938, *p* < 0.001).

## 4. Discussion 

The aim of this study was to investigate the palatability of an ORS at various stages of exercise in the heat. The main sensory finding was the change in predictors of overall liking as a function of exercise, with liking of saltiness becoming the most important factor for overall liking of all drinks post-exercise. 

### 4.1. Liking of Saltiness

Of the three beverages used in the current study, the ORS had the highest NaCl concentration (2.6 g/L; 45 mmol/L) and, not surprisingly, received the lowest liking of saltiness of the fluids tested at all time points. Liking of saltiness did not change at any time point throughout exercise for any of the drinks; although, after 60 min of exercise, liking of saltiness for the ORS increased by a median (25, 75 percentiles) of 20% (0.0, 100%; *p* < 0.05), perhaps indicating an increase in palatability to the high salt concentration. These findings are consistent with a study investigating the dose-response effects of drink Na content (0, 18, 30, 40, and 60 mmol/L) on sensory perception and palatability in a group of trained athletes [26]. Although no differences in salt perception were observed as a function of exercise, participants became more accepting of the drink containing the highest Na concentration (60 mmol/L; 1.4 g/L) while in an exercise context, as reflected by significant increases in overall acceptance and liking of saltiness compared to the sedentary condition [26]. After a 7 h water and Na-depletion period involving 8 x 30-min bouts of cycling exercise in the heat (35 °C), a significant increase in palatability to hypertonic NaCl (≥300 mmol/L; 17.5 g/L) solutions was observed in a group of healthy volunteers [25]. In contrast, after only 30 min of cycling at 50% VO_2max_, there were no differences in salt thresholds compared to before exercise [22]. Thus, the duration and intensity of exercise, and extent of fluid loss appears to determine salt preference and liking [25]. 

### 4.2. Liking of Sweetness

While the TS and PL showed a higher liking of sweetness than the ORS at all time points, sweetness liking did not change for any of the drinks throughout exercise in the current study. Similarly, Appleton [21] found no changes in liking of sweetness for a range of fluids varying in energy, electrolyte content, and osmolality throughout 60 min of exercise; there was, however, an increase in sweetness ratings of all drinks from pre- to post-exercise. In contrast, an increase in liking of sweetness has been reported following 30 min of moderate exercise in a group of students [22,23]. Following a half marathon, an increase in liking of sweetness was observed accompanied by an increase in the degree of physical fatigue [35]. Narukawa et al. [35] attributed this CHO-seeking behavior to the need to replenish glycogen stores with exercise, thus supporting the theory of physiological usefulness. However, this was not the case following a 12-h mountain hike with no significant change in liking of sweetness of a low (100 mmol/L) and high (300 mmol/L) sucrose-containing solution [36]. It is likely, however, that exercisers could easily distinguish between the two distinct sucrose concentrations, thus potentially impacting results [36]. Interestingly, this increase was not observed for fluids sweetened with the artificial sweetener saccharin, perhaps due to its synthetic nature and inability to provide energy [23]. Furthermore, another study identified only a weak correlation between sweetness and overall liking which increased throughout 60 min of moderate–high-intensity exercise for all CHO-containing drinks [37]. These discrepancies may indicate that other factors are contributing to the overall hedonic response, such as liking of saltiness and thirst-quenching ability. In the current study, both of these variables (saltiness liking and thirst-quenching ability) were identified as important predictors of overall liking of the ORS post-exercise (R^2^ = 0.938, *p* < 0.001). 

### 4.3. Osmolality

In the current study, there were no significant changes in sensory variables for the PL which had the lowest osmolality and received similar sensory ratings from rest and throughout 60 min of exercise. Furthermore, while there was a significant increase in saliva osmolality and saliva total protein concentration, our participants only reached a BML of 1.36 ± 0.39% after 60 min of exercise, and thus did not achieve the threshold for clinical “dehydration” (≥2% BML) [38,39]. Perhaps sensory changes may have occurred following fluid loss to this extent (i.e., >2.0% BML). A similar study observed an increase in pleasantness ratings for the fluids with the lowest osmolality (1.7 and 3.4% CHO) immediately after exercise [21]; this effect was most pronounced in participants who lost the greatest amount of sweat (1% BML vs. 0.4% BML), thus suggesting that water balance is more important than electrolyte or energy intake—especially in those who sweat more during exercise [21]. However, in the Appleton [21] study, the exercise protocol was not controlled (i.e., the method and intensity), thus impacting the reliability and validity of these results. 

### 4.4. Thirst Quenching Ability

While the current study did not find any changes in thirst-quenching ability for any of the drinks, there was an increase in percent thirst-quenching ability ratings for the ORS after 60 min of exercise (*p* < 0.05). Furthermore, thirst-quenching ability, together with liking of saltiness, was identified as a key predictor of overall liking of the ORS post-exercise (R^2^ = 0.938, *p* < 0.001). 

### 4.5. Overall Liking

In the current study, the TS was the most-preferred and the ORS was the least-liked beverage at all time points; nonetheless, research points to a shift during exercise in which the hedonic value of a beverage can change dramatically from sedentary to exercise conditions [15]. Substantial increases in acceptability of the least-liked beverage have been observed over 180 min of exercise at 70–75% HR_max_ [15]. Similarly, palatability ratings of hypertonic NaCl solutions (≥300 mmol; 17.5 g/L NaCl) have been shown to increase throughout thermal and exercise-induced fluid loss [25]. Ali et al. [37] found overall liking to increase with exercise for all drinks tested (including water), suggesting that replenishment of lost fluids during exercise is the most important factor in determining overall liking. In contrast, there were no changes in overall liking for any of the drinks in the current study; instead, liking of saltiness and thirst-quenching ability became the most important predictors of overall liking following 60 min of exercise (R^2^ = 0.938, *p* < 0.001). Furthermore, after 60 min of exercise, the ORS showed a significant increase in percent liking of saltiness and thirst-quenching ability ratings, perhaps indicating an enhanced palatability to saltiness. These findings are consistent with the theory of physiological usefulness in which exercise elicits an increase in liking of saltiness as salt is progressively lost through sweat. Therefore, it seems reasonable to expect a further improvement in overall liking of the ORS among highly trained athletes (relative to recreational exercisers) who are likely to exercise for a longer duration, at a higher intensity, and with larger sweat losses. 

### 4.6. Limitations

This study did not reach the critical value for exercise-induced dehydration (≥2% body mass loss) [38,39] with a body mass loss of only 1.36 ± 0.39% following 3 × 20-min bouts of moderate intensity exercise in the heat. Furthermore, plasma osmolality was not measured, and the gold standard for assessing hydration status was considered [39]. We used aural temperature to indicate core temperature rather than more accepted methods such as rectal or oesophageal temperature. Participants were only recreationally active; thus, findings may not be reflective of the highly trained athletic population.

## 5. Conclusions

In this study, we showed that although the ORS was identified as the least-liked beverage at rest, participants’ liking of its saltiness and thirst-quenching ability significantly increased from pre- to post-exercise. Furthermore, liking of saltiness and thirst-quenching ability became the most important predictors of overall liking post-exercise. Since the electrolyte content of traditional sports drinks is insufficient for most athletes undertaking prolonged exercise in the heat, and because the same beverage may not be suitable for all stages of exercise due to the physiological changes occurring during exercise, the findings from this study may be an important step for the formulation of sports beverages, namely, beverages that meet both physiological and perceptual requirements for athletes undertaking prolonged exercise in the heat. 

## Figures and Tables

**Table 1:** Composition of the drinks used for this study. Carbohydrate content (g/L and mmol/L), electrolyte content (g/L and mmol/L), and flavoring (g/L) for each drink: Oral rehydration solution (ORS), placebo (PL), traditional sports drink (TS) prepared for this study.

Ingredient	Oral Rehydration Solution (ORS)	Sports Drink (TS)	Placebo (PL)
Glucose (mmol/L)	72	0	0
Sucrose (mmol/L)	23.4	172.4	0
Fructose (mmol/L)	16.6	0	0
Maltodextrin (mmol/L)	0	31.7	0
Sodium (mmol/L)	52.25	4.79	0
Potassium (mmol/L)	30.06	0.46	0
Citrate (mmol/L)	12.00	1.45	0
Chloride (mmol/L)	71.3	4.79	0
Osmolarity (mmol/L)	278	216	0

**Table 2:** Physiological and perceptual responses throughout exercise. ^†^ Values are means ± SD. ^⊥^ Values are geometric means (95% CI). ^‡^ Values are medians (25, 75 percentiles). * Significant change at 60 min (*p* < 0.05). ** Significant change at 60 min (*p* < 0.001).

Exercise Sample Time	0 min	20 min	40 min	60 min
Body mass (kg) ^†^	69.01 ± 8.63	68.81 ± 8.59	68.51 ± 8.54	68.07 ± 8.49
Body mass loss (kg) ^†^	-	0.20 ± 0.11	0.50 ± 0.18	0.94 ± 0.30 **
Body mass loss (%) ^†^	-	0.29 ± 0.16	0.72 ± 0.24	1.36 ± 0.39 **
Saliva osmolality (mOsmol/kg) ^⊥^	85 (78, 92)	91 (85, 98)	101 (94, 109)	113 (102, 124) **
Saliva protein concentration (mg/mL) ^⊥^	1.27 (1.06, 1.49)	1.59 (1.27, 1.94)	2.33 (1.90, 2.83)	3.17 (2.51, 3.96) **
Urine specific gravity ^‡^	1.014 (1.01, 1.02)	-	-	1.017 (1.014, 1.025) *
Urine osmolality (mOsmol/kg) ^‡^	516 (227, 876)	-	-	586 (476, 796)
Urine colour ^‡^	3.0 (1.0, 4.0)	-	-	4.0 (3.0, 7.0) **
Aural temperature (°C) ^†^	36.1 ± 0.6	37.4 ± 0.2	38.3 ± 0.4	38.4 ± 0.6 *
Heart rate (bpm) ^†^	82.9 ± 19.0	152.4 ± 19.8	160.7 ± 22.8	169.7 ± 20.2 **
Ratings of perceived exertion ^†^	-	13.2 ± 1.5	15.6 ± 1.3	17.2 ± 1.3 **
Felt arousal scale ^†^	-	3.5 ± 1.0	3.8 ± 1.0	3.7 ± 1.2
Feeling Scale ^†^	-	1.3 ± 1.9	−0.6 ± 1.9	−1.4 ± 1.8 **
Thermal comfort ^†^	-	5.1 ± 1.3	6.7 ± 1.1	7.5 ± 1.4 **
Work rate (W) ^†^	-	98 ± 23	97.7 ± 24	96 ± 24

## Data Availability

Data is contained within the article.
